# Beyond the Scale: Effects of Maternal Obesity on Embryo Morphokinetics and IVF Outcomes

**DOI:** 10.3390/jcm15062182

**Published:** 2026-03-12

**Authors:** Nir Roguin, Medeia Michaeli, Diana Polotov, Einat Shalom-Paz

**Affiliations:** 1 Joyce and Irving Goldman Medical School, Faculty of Health Sciences, Ben-Gurion University of the Negev, Be’er Sheva 8410501, Israel; 2 IVF Unit, Department of Obstetrics & Gynecology, Hillel Yaffe Medical Center, Hadera 3810101, Israel; 3 Ruth and Bruce Rappaport Faculty of Medicine, The Technion-Israel Institute of Technology, Haifa 3200003, Israel

**Keywords:** clinical pregnancy rate, body mass index, live birth rate, pregnancy complications, morphokinetics, time-lapse imaging

## Abstract

**Background:** Does maternal body mass index (BMI) influence embryo morphokinetics in fresh embryo transfer cycles, and how does this relate to clinical outcomes and obstetric complications? **Methods**: A retrospective cohort study was conducted on 2238 fresh embryo transfer (ET) cycles, categorized into four BMI groups: underweight, normal weight, overweight, and obese. Baseline characteristics, stimulation parameters, hormonal profiles, morphokinetic data, and pregnancy and delivery outcomes were analyzed. **Results**: Higher BMI was associated with more anovulatory infertility and greater endometrial thickness. Peak estradiol and estradiol-to-oocyte ratios declined progressively with increasing BMI, despite preserved oocyte yield and embryo quality scores. Interestingly, the underweight group exhibited a significantly distinct biphasic morphokinetics developmental pattern compared with the overweight and obese groups. Pregnancy rates, including clinical and live birth, did not differ significantly across BMI groups. However, obese women had markedly higher cesarean section rates (51.9% vs. ~25–28% in other groups) and a non-significant trend toward more gestational diabetes. Other perinatal outcomes, such as preeclampsia and preterm birth, were not significantly different. **Conclusions**: In fresh IVF cycles, a higher BMI does not impair pregnancy achievement but is linked to altered hormonal response and increased obstetric risk, particularly cesarean delivery. These findings highlight the importance of preconception counseling and targeted obstetric management for women with elevated BMI undergoing fresh ET.

## 1. Introduction

Obesity affects over 40% of reproductive-aged women worldwide [1]. It is consistently linked to poorer reproductive outcomes, including ovulatory dysfunction and elevated risks of gestational diabetes (GDM), hypertensive disorders, and cesarean delivery [2]. In IVF, concerns persist regarding how maternal body mass index (BMI) impacts both ovarian response and embryo development.

Findings regarding BMI and IVF success remain inconsistent. Some studies report lower oocyte yield and reduced live birth rates with elevated BMI [3], citing altered steroidogenesis and inflammation [4]. Conversely, others suggest success rates are comparable when high-quality embryos are transferred, implying age or endometrial factors may be more decisive [5].

Most studies rely on static embryo assessment, providing limited insight into early embryogenesis. Time-lapse incubator (TLI) systems allow for continuous monitoring of cleavage timing and blastocyst formation [6]. However, few studies have utilized TLI to examine how maternal BMI affects specific morphokinetics [7].

This study aimed to assess the association between maternal BMI and embryo morphokinetics and determine their correlation with clinical and obstetric outcomes. Primary outcomes included cleavage timing and embryo quality (KIDscore), while secondary outcomes focused on pregnancy, delivery, and complication rates.

## 2. Methods

This retrospective cohort study at Hillel Yaffe Medical Center (January 2018–December 2024) included fresh IVF cycles using autologous oocytes, TLI culture, and fresh embryo transfer (ET). We excluded severe male factor (few motile cells) and uterine abnormalities (septate, T-shaped, unicornuate, or markedly myomatous uteri), though mild-to-moderate male factor was included.

To maximize power, fertility preservation cycles were included for oocyte yield analysis; however, pregnancy rates were calculated strictly per fresh ET to avoid bias from elective cryopreservation. The Institutional Review Board approved the study (0026-20-HYMC), and informed consent was waived.

Patients were categorized by BMI: underweight (<18.5 kg/m^2^), normal weight (18.5–24.99 kg/m^2^), overweight (25–29.99 kg/m^2^), and obese (≥30 kg/m^2^) per CDC criteria [8].

### 2.1. Ovarian Stimulation and ET

All patients used tailored GnRH antagonist protocols [9]. Gonadotropin dosing (recombinant FSH and/or purified HMG) was individualized based on age, BMI, and ovarian reserve. Serum E2, progesterone, and LH were measured at every follow-up alongside transvaginal ultrasound (TVUS).

Antagonists (0.25 mg Cetrorelix (Merck Serono, Darmstadt)/Ganirelix (Organon, Oss, The Netherlands)) were initiated when follicles reached ≥12 mm. Once leading follicles were ≥17 mm, ovulation was triggered with recombinant hCG and/or 0.2 mg GnRH agonist, followed by retrieval 36 h later and fertilization via IVF or ICSI [10].

ET occurred on Day 3 or 5. Luteal support included oral dydrogesterone combined with vaginal progesterone gel or tablets.

ET decisions were based on oocyte yield, E2 levels (to mitigate OHSS risk), and embryo quality. Single ET was standard; two embryos were transferred only after >3 unsuccessful cycles. All embryos were cryopreserved if endometrial abnormalities were detected or if progesterone rose prematurely.

### 2.2. Evaluation of Time-Lapse Images

Embryos were assessed via TLI (EmbryoViewer, Unisense FertiliTech, Aarhus, Denmark), which provided continuous image acquisition without disturbing culture conditions. Only 2PN embryos were included; for Day 3 transfers, data were censored at incubator removal, while later milestones were derived from embryos cultured until Day 5/6.

Arrested embryos were analyzed until the point of arrest. Recorded timings included tPB2, tPNa, tPNf, and cleavage stages t2, t3, t4, t5, and t8. Cell cycle intervals (Cc2, Cc3), synchrony (s2, s3), and KIDscore, integrating morphokinetic and morphological data for implantation potential, were assessed. Morphokinetic analysis was performed at the individual oocyte level.

### 2.3. Outcome Measures

Biochemical pregnancy was confirmed via serum β-hCG 12 days post-transfer, and clinical pregnancy via TVUS at 6 weeks. All pregnancies were followed until delivery to record maternal/fetal complications and mode of delivery. Clinical outcome analyses were performed at the cycle level, with one observation per fresh embryo transfer.

### 2.4. Statistical Analysis

Analysis was performed using SPSS version 29. Normality was assessed using the Shapiro–Wilk test. Continuous variables were compared using one-way ANOVA or the Kruskal–Wallis test, as appropriate, with post hoc pairwise comparisons adjusted by Bonferroni correction. Independent samples *t*-test and Mann–Whitney U test were used for comparisons between two groups. Categorical variables were analyzed using Pearson’s chi-square test or Fisher’s exact test.

To account for the non-independence of multiple embryos originating from the same patient, morphokinetic parameters were analyzed using a linear mixed-effects model (LMM) with patient as a random effect. The fixed effects included BMI category, developmental timepoint, and their interaction. Pairwise comparisons between BMI groups at each timepoint were performed with Bonferroni correction.

Given the large number of comparisons across BMI categories and multiple outcome variables, the Benjamini–Hochberg false discovery rate (FDR) procedure was applied to adjust for multiple testing. All reported *p*-values reflect FDR-adjusted values, with significance set at q < 0.05. Multivariable logistic regression was used to identify independent predictors of clinical pregnancy, adjusting for BMI category, maternal age, male factor infertility, endometrial thickness, and LH and progesterone levels at trigger.

### 2.5. Disclosure of Generative AI

The authors used Gemini 3 (Google, California, USA, January 2026, as available online) to perform spellchecking and to improve the language, flow, and structural clarity of the manuscript. Following this, all authors manually reviewed, edited, and verified the generated text to ensure it accurately reflects the study’s data and findings. The authors take full responsibility for the final content and the integrity of the work.

## 3. Results

A total of 2238 fresh IVF cycles were stratified by BMI: underweight (*n* = 102), normal weight (n = 1001), overweight (n = 560), and obese (n = 575).

### 3.1. Baseline Characteristics

Maternal age was comparable across groups (*p* = 0.054). Infertility etiologies differed significantly: male factor (*p* = 0.009), tubal/uterine factor (*p* = 0.008), and anovulation (*p* < 0.001) were significantly different. Notably, anovulation was absent in underweight women but reached 15.7% in the obese group. Endometrial thickness increased progressively with BMI, from 9.34 ± 2.43 mm in underweight to 10.05 ± 2.44 mm in obese patients (*p* < 0.001) (Table 1).

A sub-analysis of fertility preservation cycles is presented in Supplementary Table S1. These cycles showed similar trends in ovarian response relative to BMI. Importantly, inclusion of these patients primarily contributed to the analysis of oocyte yield.

### 3.2. Cycle Parameters

Oocyte yield (*p* = 0.98), the number of fertilized oocytes (*p* = 0.43), and fertilization rates (59–60%; *p* = 0.23) were similar across all BMI categories. In the fertility preservation subgroup, oocyte yield showed a trend toward higher retrieval in the normal weight group compared with the obese group, though this did not reach significance after FDR correction (q = 0.064).

Peak estradiol levels on trigger day decreased significantly with increasing BMI, with median levels ranging from 1505 pg/mL in underweight to 627 pg/mL in obese patients (normal weight vs. obese, q < 0.001). The estradiol-to-oocyte ratio followed a similar significant decline (normal weight vs. obese q = 0.006; normal weight vs. overweight q = 0.037).

KIDScore values were numerically highest in the underweight group (4.62 ± 0.79) and lowest in the normal weight group (4.29 ± 1.1), but the difference did not remain significant after FDR correction (q = 0.064).

The distribution of cleavage-stage (Day 3) versus blastocyst-stage (Day 5) transfers was comparable across all study groups (*p* = 0.13) (Table 2).

### 3.3. Embryo Development

A linear mixed-effects model with patient as a random effect confirmed a significant BMI × developmental timepoint interaction (*p* < 0.001), indicating that embryo developmental trajectories differ across BMI categories. All morphokinetic comparisons that were significant in the original analysis remained significant after Benjamini–Hochberg FDR correction.

Early cleavage occurred sooner in underweight patients. tPNf was significantly shorter in the underweight group (23.8 ± 3.0 h) compared to the normal weight (P1 = 0.028) and obese groups (P3 = 0.027). Similarly, t2 was reached earlier in the underweight group (26.68 ± 3.52 h) than in the normal weight (P1 = 0.005) and obese (P3 = 0.035) cohorts. Normal weight embryos also reached t2 later than overweight embryos (P4 = 0.039). Acceleration in the underweight group persisted through t3 (37.1 ± 5.8 h vs. 38.4–38.6 h; P1 = 0.008, P3 = 0.022), where overweight embryos were also significantly faster than normal weight (P4 = 0.005).

This trend reversed at the 5-cell stage (t5), with overweight patients reaching cleavage earlier (P4 = 0.019). By t8, embryos from overweight women were the fastest (58.8 ± 10.3 h), significantly outpacing the normal weight (P4 = 0.019) and obese (P6 = 0.036) cohorts.

**Figure 1:**
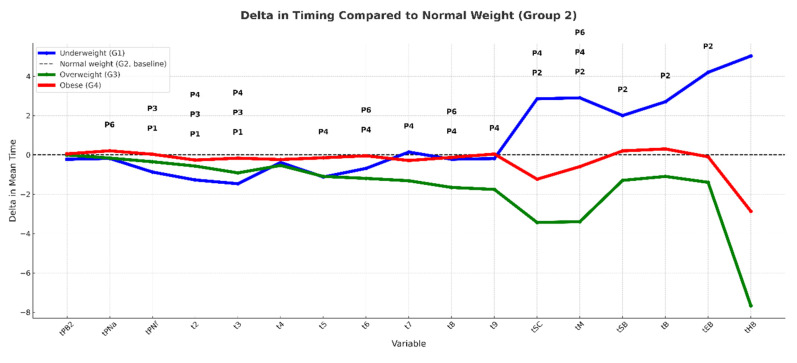
Delta timing of embryo developmental events according to maternal BMI category. Graph depicts mean ± SD differences (Δ hours post-insemination) in morphokinetic parameters relative to the normal weight group, derived from time-lapse imaging of embryos in fresh IVF cycles. Events include pronuclear fading (tPNf), cleavage to 2-, 3-, 5-, and 8-cell stages (t2, t3, t5, t8), morula formation (tM), start of blastulation (tSB), and full blastocyst formation (tB). Negative Δ values indicate earlier occurrence compared with normal weight, positive values indicate delay. Embryos of the underweight group exhibited earlier early cleavage events, whereas embryos of the obese group showed later morula and blastocyst formation. BMI, body mass index; Δ, delta difference; tPNf, time to pronuclear fading; tM, morula formation; tSB, start of blastulation; tB, blastocyst formation. Post-hoc *p*-value notation: P1 = underweight vs. normal, P2 = underweight vs. overweight, P3 = underweight vs. obese, P4 = normal vs. overweight, P5 = normal vs. obese, P6 = overweight vs. obese.

At the blastocyst stage (tB), development remained faster in overweight women (107.6 ± 8.89 h) compared to underweight patients (P2 = 0.019), indicating faster late-stage development in higher BMI groups (Table 3). Figure 1 illustrates these deviations from the normal weight mean; negative Δ values indicate earlier development, while positive values indicate delay (Figure 1).

### 3.4. Pregnancy Outcomes

Rates of positive β-hCG, clinical, ongoing, and live birth were not significantly different across groups (clinical pregnancy: 33.3% underweight vs. 29.9% obese; *p* = 0.93) (Table 2).

**Figure 2:**
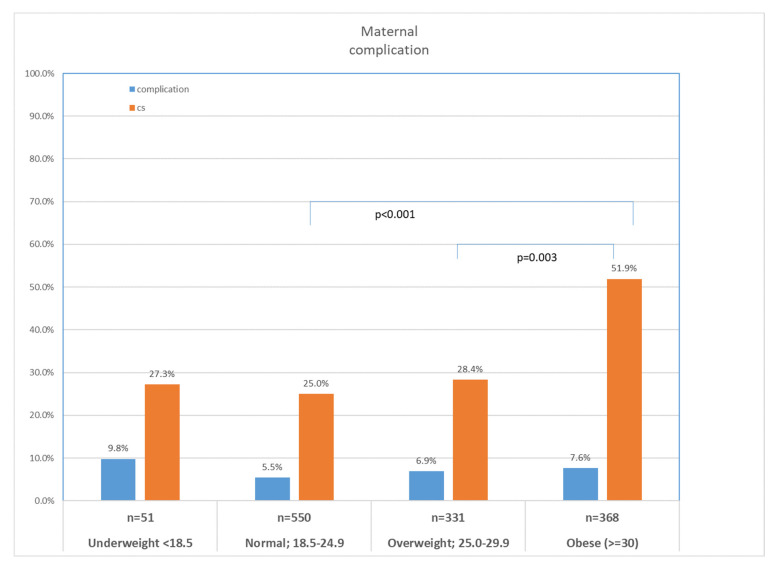
Caesarean section rate and overall obstetric complication rate by maternal BMI category in live births following fresh embryo transfer. *p*-values shown are unadjusted; after Benjamini–Hochberg correction (FDR < 0.05), both comparisons remained statistically significant (adjusted *p* = 0.001 and *p* = 0.01, respectively).

Cesarean section (CS) rates differed significantly (*p* < 0.001), with obese patients reaching 51.9% compared to 27.3% in underweight and 25.0% in normal weight women. Gestational diabetes (GDM) was more frequent in overweight (19.1%) and obese (19.4%) patients than normal weight (10.3%) and underweight (0%) women (*p* = 0.060). Mean birthweight showed a non-significant increasing trend from 2734 ± 542 g in underweight to 3156 ± 664 g in obese patients (Table 4) (Figure 2).

### 3.5. Predictors of Clinical Pregnancy and Live Birth

In order to predict clinical pregnancy, we compared baseline characteristics. We found that clinical pregnancy was associated with younger age (32.7 ± 5.8 vs. 35.4 ± 6.5 years; *p* < 0.001), anovulatory (*p* = 0.013) or male factor infertility (*p* = 0.038), and higher oocyte yield (10.0 ± 6.3 vs. 8.7 ± 6.0; *p* < 0.001).

For live birth, BMI was similar between groups (26.7 ± 6.12 vs. 26.5 ± 6.22; *p* = 0.75), as was BMI distribution (*p* = 0.62). Women with live birth were younger (32.3 ± 5.6 vs. 34.5 ± 6.32; *p* = 0.002), had slightly lower basal FSH (*p* = 0.02), and fewer previous transfers (2.49 ± 2.45 vs. 3.22 ± 2.94; *p* = 0.028) (Table 5).

### 3.6. Multivariable Regression

Based on the univariate, we conducted a multivariate logistic regression to predict clinical pregnancy. Independent predictors of clinical pregnancy were endometrial thickness (aOR 1.118, 95% CI: 1.018–1.229; *p* = 0.02) and serum progesterone at trigger (aOR 1.057, 95% CI: 1.038–1.077; *p* < 0.001). Adjusted for confounders, BMI was not significantly associated with pregnancy, and neither overweight (aOR 1.015; *p* = 0.962) nor obesity (aOR 1.432; *p* = 0.201) demonstrated significant impacts.

For live birth, maternal age remained a significant independent predictor (aOR 0.925, 95% CI: 0.868–0.986; *p* = 0.016). BMI (aOR 1.024; *p* = 0.353) and transfer type (single/double) were not significant. Oocyte yield was positively associated with live birth (aOR 1.079, 95% CI: 1.013–1.150; *p* = 0.019) (Table 6).

## 4. Discussion

In this cohort of over 2200 fresh ET cycles, maternal BMI influenced hormonal profiles and embryo developmental kinetics but did not compromise pregnancy outcomes. Live birth and clinical pregnancy rates were comparable across BMI groups, suggesting preserved implantation potential in the fresh transfer setting. However, obese women had markedly higher cesarean rates, highlighting BMI as a determinant of perinatal course rather than conception success.

### 4.1. Baseline Characteristics

The higher prevalence of anovulatory infertility with increasing BMI is consistent with obesity-mediated ovulatory dysfunction [11]. The thicker endometrium observed in higher BMI groups may reflect peripheral aromatization of androgens to estrone in adipose tissue [12], though it is worth noting that endometrial receptivity could be impaired by altered inflammatory and cytokine profiles, potentially offsetting this anatomical advantage [5,13].

### 4.2. Cycle Parameters and Hormonal Response

Although oocyte yield was preserved across BMI groups, peak estradiol and estradiol-to-oocyte ratios decreased with increasing BMI, which may reflect reduced granulosa cell responsiveness and altered steroidogenesis [14,15,16]. Despite these hormonal disparities, pregnancy rates remained comparable, suggesting that competent embryos can be obtained even with suboptimal hormonal markers [17]. The relatively young and uniform age of our cohort likely contributed to this finding, as age remains the dominant determinant of IVF success [18].

### 4.3. Morphokinetic Trends and Clinical Outcomes by BMI

A linear mixed-effects model confirmed a significant BMI interaction with developmental timepoint (*p* < 0.001), and all individual pairwise comparisons remained significant after Benjamini–Hochberg FDR correction, supporting the robustness of the observed morphokinetic patterns.

Morphokinetic analysis revealed a biphasic pattern (Figure 1): embryos from underweight women reached early cleavage milestones faster (shorter tPNf, t2, t3), but this advantage reversed around the 4-cell stage. From t5 onward, overweight women’s embryos progressed through later stages (tM, tSB, tB) faster than those of normal weight and obese groups.

### 4.4. Oocyte Quality and Maternal BMI

It is well established that embryonic genome activation (EGA) occurs between the 4- and 8-cell stages, and that cleavage before this transition depends largely on oocyte quality [19]. One possible explanation for the early acceleration in underweight women is that their oocytes may be less susceptible to lipotoxic damage; studies in animal models have shown that oocytes from obese individuals display mitochondrial abnormalities and reduced membrane potential [20], and human data suggest lower mitochondrial DNA expression in the cumulus cells of overweight women [21,22]. However, we did not directly measure lipotoxicity markers or mitochondrial function, so this interpretation remains speculative.

The post-EGA catch-up in overweight embryos is also intriguing. At the blastocyst stage, metabolic demand rises and fatty acid β-oxidation becomes an important ATP source [23,24]. Follicular fluid triglyceride levels have been reported to be highest in overweight women [25], which could theoretically provide greater lipid substrate for later-stage development. However, this hypothesis requires direct validation with paired metabolomic and morphokinetic data.

Importantly, these phase-specific differences appear to converge by transfer time: underweight embryos’ early advantage is offset by post-EGA deceleration, while overweight embryos catch up. Laboratory selection of the best-performing embryo further compresses these kinetic differences. In our cohort, endometrial thickness—not BMI—was the independent predictor of clinical pregnancy. These findings are consistent with Weinerman et al. [26] and Rubio et al. [27], and align with the recent work of Younes et al. [28], who similarly reported that BMI-related differences in embryo development did not translate to impaired clinical outcomes. Taken together, these data suggest that once embryos reach blastocyst competence, the uterine environment may matter more than subtle developmental timing differences.

### 4.5. Obstetric Outcomes

The sharp rise in cesarean delivery among obese women is consistent with the literature linking a higher BMI to longer labor, macrosomia, and clinician preference for surgical delivery [29]. The trend toward higher GDM rates in overweight and obese groups (19.1% and 19.4% vs. 10.3% in normal weight; *p* = 0.06) aligns with established pathophysiology—including insulin resistance, chronic low-grade inflammation, and altered adipokine profiles—though our study may have been underpowered to detect a statistically significant difference given the relatively small number of deliveries per group. Similarly, the preeclampsia trend (*p* = 0.08) is consistent with shared pathophysiological mechanisms between obesity and preeclampsia, including endothelial dysfunction and oxidative stress [30]. It is possible that obesity-related metabolic dysfunction compounds the already elevated metabolic demands of IVF pregnancies. Other perinatal outcomes did not differ significantly, which may reflect both sample size limitations and the effectiveness of antenatal surveillance in this cohort [31,32].

In our multivariable model, endometrial thickness and progesterone at trigger were independent predictors of clinical pregnancy, in line with previous studies [33,34], while BMI category was not significant after adjustment. These findings suggest that BMI influences reproductive outcomes primarily through hormonal and endometrial modulation—including impaired granulosa responsiveness [14,15,16] and increased adipose-driven estrogen metabolism [12]—rather than through direct impairment of implantation potential. This mechanistic pattern aligns with our morphokinetic data: although embryo development differed across BMI groups, these differences did not translate into reduced pregnancy rates once transfer occurred.

Nonetheless, the association between elevated BMI and increased cesarean delivery rates, along with a trend toward greater metabolic complications, underscores the importance of preconception optimization and targeted antenatal care. It is worth noting that this cohort was uniformly young, which may limit the ability to detect age–BMI interactions and partially explain why BMI did not independently predict clinical pregnancy.

Strengths include the large single-center cohort with standardized TLI culture, broad BMI spectrum, adjustment for confounders, and follow-up through delivery. Focusing on fresh transfers avoids cryopreservation confounding.

Limitations include the retrospective, single-center design with inherent selection biases. Embryo ploidy was not assessed by preimplantation genetic testing (PGT-A), and the potential effect of BMI on aneuploidy rates cannot be excluded. Additionally, residual confounding from unmeasured variables such as diet, physical activity, and environmental exposures cannot be ruled out [35,36].

## 5. Conclusions

In conclusion, in this large cohort of fresh IVF cycles, maternal BMI influenced baseline characteristics, hormonal profiles, and delivery mode but did not reduce pregnancy rates. The most pronounced risk associated with obesity was a higher cesarean section rate, with a trend toward increased metabolic complications. Effective embryo selection and preserved implantation potential may explain the similar pregnancy outcomes, but the elevated obstetric risk profile underscores the need for individualized counseling and proactive perinatal management in this population.

## Figures and Tables

**Table 1:** Baseline characteristics of fresh embryo transfer cycles according to maternal BMI category.

Characteristic	Underweight (n = 102)	Normal Weight (n = 1001)	Overweight (n = 560)	Obese (n = 575)	*p*-Value
BMI (kg/m^2^)	17.5 ± 0.9	21.8 ± 1.6	27.2 ± 1.5	34.9 ± 3.8	–
Age (years)	33.6 ± 5.5	34.9 ± 6.0	34.2 ± 6.2	34.7 ± 6.3	0.054
Cause of infertility					
Male factor	24 (24.0%)	265 (28.1%)	192 (34.3%)	175 (31.6%)	0.009
Anovulation	0 (0%)	37 (3.9%)	24 (4.5%)	87 (15.7%)	<0.001
Tubal/uterine factor	17 (17.7%)	77 (8.2%)	63 (11.7%)	55 (9.9%)	0.008
Endometriosis	3 (3.1%)	25 (2.6%)	7 (1.3%)	5 (0.9%)	0.053
Unexplained	39 (40.6%)	401 (42.5%)	187 (34.8%)	200 (36.1%)	0.01
Fertility preservation	14 (14.6%)	139 (14.7%)	65 (12.1%)	32 (5.8%)	<0.001
Basal FSH (IU/L) ^†^	12.8 ± 2.6	8.9 ± 5.5	8.6 ± 3.7	7.6 ± 3.2	<0.001
Basal LH (IU/L) ^†^	6.4 ± 3.1 (n = 92)	6.4 ± 3.9	5.2 ± 4.3	5.5 ± 3.8	0.03
Last endometrial thickness (mm)	9.34 ± 2.43	9.39 ± 2.55	9.76 ± 2.53	10.05 ± 2.44	<0.001
Previous IVF cycles median [IQR]	2 [1–4]	2 [1–4]	2 [1–3]	2 [1–4]	0.32

Values are presented as mean ± standard deviation (SD), median [interquartile range (IQR)], or n (%). BMI = body mass index (kg/m^2^); FSH = follicle-stimulating hormone; LH = luteinizing hormone; mm = millimeters; IVF = in vitro fertilization; IQR = interquartile range; SD = standard deviation. † Basal hormone levels measured prior to stimulation onset; values available for a subset of patients.

**Table 2:** Cycle parameters, hormonal profiles, and pregnancy rates by maternal BMI category.

Parameter		Underweight (n = 102)	Normal Weight (n = 1001)	Overweight (n = 560)	Obese (n = 575)	BH-Adjusted *p*
IVF Drug						
Menopur use		51 (52.6%)	520 (54.2%)	286 (53.0%)	305 (55.1%)	0.96
Pergoveris use		46 (47.4%)	440 (45.8%)	254 (47.0%)	249 (44.9%)	0.96
Last estradiol (pg/mL)		1505 [275–2953]	1184 [269–2629]	900 [220–1921]	627 [197–1643]	P3 = 0.072 P5 = 0.0008
Last LH (IU/L)		2.14 [0.64–5.33]	3.14 [1.61–5.22]	3.12 [1.80–5.23]	3.65 [2.27–5.25]	0.091
Right ovary mean No. follicles ≥ 14 mm		4.38 ± 2.79	4.08 ± 2.60	3.96 ± 2.65	4.06 ± 2.87	0.862
Left ovary mean No. follicles ≥ 14 mm		4.37 ± 2.42	4.00 ± 2.59	3.78 ± 2.43	3.75 ± 2.47	0.208
Retrieved oocytes (all cohort)		10.1 ± 6.5	10.3 ± 8.3	10.4 ± 8.3	10.2 ± 8.0	0.98
Retrieved oocytes (fertility preservation cohort)		12.1 ± 5.8	13.5 ± 10.4	10.2 ± 8.03	8.66 ± 7.2	0.064
Estradiol/oocyte ratio (pg/mL)		190.4 [47.3–284.5]	171.5 [53.2–274.5]	126.5 [33.7–237.1]	117.4 [33.5–206.8]	P5 = 0.006 P4 = 0.037
Fertilized oocytes (2PN)		6.11 ± 3.9	6.21 ± 5.05	6.46 ± 5.32	5.92 ± 4.88	0.573
Fertilization rate		0.60 ± 0.20	0.60 ± 0.22	0.59 ± 0.22	0.87 ± 0.23	0.335
KIDScore (mean ± SD)		4.62 ± 0.79	4.29 ± 1.10	4.43 ± 0.98	4.35 ± 1.07	0.064
Cryopreserved embryos		3.28 ± 2.42	3.48 ± 2.87	3.49 ± 2.90	3.00 ± 2.26	0.2
No. of embryos transferred	1	26 (72.2%)	293 (69.0%)	185 (70.0%)	199 (72.6%)	–
	2	10 (27.8%)	121 (28.5%)	69 (26.0%)	67 (24.5%)	
Day		N = 315	N = 1948	N = 1248	N = 1455	0.208
Day 3		221 (70.2%)	1419 (72.8%)	894 (71.6%)	1092 (75.1%)	
Day 5		94 (29.8%)	529 (27.2%)	354 (28.4%)	363 (24.9%)	
**Pregnancy outcome**						
Positive hCG		20/51 (39.2%)	212/550 (38.5%)	130/331 (39.3%)	136/368 (37.0%)	NS
Clinical pregnancy per transfer (%)		17/51 (33.3%)	167/550 (30.4%)	105/331 (31.7%)	110/368 (29.9%)	NS
Miscarriage per transfer (%)		3/51 (5.8%)	45/550 (8.2%)	25/331 (7.6%)	26/368 (7.0%)	NS

Values are mean ± SD, median [IQR], or n (%). BMI = body mass index; 2PN = two pronuclei; KIDScore = known implantation data score; E2 = estradiol; LH = luteinizing hormone; pg/mL = picograms per milliliter; IU/L = international units per liter; hCG = human chorionic gonadotropin. *p*-values from ANOVA, Kruskal–Wallis, or chi-square tests as appropriate. P1 = underweight vs. normal weight, P2 = underweight vs. overweight, P3 = underweight vs. obese, P4 = normal weight vs. overweight, P5 = normal weight vs. obese, P6 = overweight vs. obese. BH—(Benjamini–Hochberg). NS = not significant

**Table 3:** Embryo morphokinetics (hours) in fresh IVF cycles according to maternal BMI category.

Variable	Group 1	Group 2	Group 3	Group 4	BH-Adjusted *p*
tPB2	3.01 ± 1.1	3.24 ± 2.12	3.23 ± 2.1	3.29 ± 2.58	0.4050
tPNa	6.962 ± 1.408	7.144 ± 2.4772	6.98 ± 2.267	7.348 ± 3.0742	P6 = 0.0394
tPNf	23.848 ± 3.027	24.724 ± 4.6473	24.371 ± 4.6529	24.752 ± 5.284	P1 = 0.0284 P3 = 0.0270
t2	26.68 ± 3.517	27.96 ± 5.678	27.388 ± 5.5205	27.694 ± 5.754	P1 = 0.0054 P3 = 0.0351 P4 = 0.0394
t3	37.09 ± 5.843	38.56 ± 6.744	37.641 ± 6.219	38.39 ± 6.5076	P1 = 0.0077 P3 = 0.0216 P4 = 0.0054
t4	40.34 ± 7.001	40.73 ± 7.56	40.19 ± 7.241	40.49 ± 7.032	0.3132
t5	50.26 ± 9.063	51.39 ± 8.804	50.29 ± 8.759	51.24 ± 8.0968	P4 = 0.0193
t6	53.75 ± 9.042	54.44 ± 9.322	53.24 ± 9.098	54.39 ± 8.423	P4 = 0.0193 P6 = 0.0368
t7	57.41 ± 10.67	57.27 ± 10.11	55.95 ± 9.807	56.98 ± 9.2	P4 = 0.0219
t8	60.244 ± 10.87	60.47 ± 11.37	58.81 ± 10.28	60.34 ± 10.92	P4 = 0.0193 P6 = 0.0368
t9	69.67 ± 11.16	69.86 ± 11.88	68.1 ± 11.386	69.9 ± 11.61	P4 = 0.0193
tSC	89.12 ± 9.169	86.27 ± 11.51	82.83 ± 12.19	85.03 ± 12.38	P2 = 0.0193 P4 = 0.0054
tM	95.9 ± 8.54	93 ± 10.41	89.6 ± 10.57	92.4 ± 10.21	P2 = 0.0014 P4 = 0.0014 P6 = 0.0077
tSB	102.5 ± 7.449	100.5 ± 8.7	99.2 ± 9.29	100.7 ± 8.55	P2 = 0.0193
tB	111.4 ± 8.09	108.7 ± 8.49	107.6 ± 8.89	109 ± 9.32	P2 = 0.0193
tEB	120.6 ± 9.5	116.4 ± 9.24	115 ± 9.82	116.3 ± 9.05	P2 = 0.0284
tHB	123.5 ± 21.28	118.47 ± 8.988	110.8 ± 6.981	115.6 ± 8.749	0.5400

Values are mean ± SD. tPB2 = time to second polar body extrusion; tPNa = time to pronucleus appearance; tPNf = time to pronucleus fading; t2–t9 = time to 2–9-cell stage; tSC = time to start of compaction; tM = time to morula; tSB = time to start of blastulation; tB = time to full blastocyst; tEB = time to expanded blastocyst; tHB = time to hatching blastocyst; SD = standard deviation. BH—(Benjamini–Hochberg). P1 = underweight vs. normal weight, P2 = underweight vs. overweight, P3 = underweight vs. obese, P4 = normal weight vs. overweight, P5 = normal weight vs. obese, P6 = overweight vs. obese.

**Table 4:** Obstetric and perinatal outcomes by maternal BMI category in live deliveries.

Outcome	Underweight < 18.5 (n = 102)	Normal 18.5–24.9 (n = 1001)	Overweight 25.0–29.9 (n = 560)	Obese ≥ 30 (n = 575)	BH Adjust *p* Value/Post Hoc
**Delivery Rate**					
Per transfer	13/51 (25.5%)	134/550 (24.4%)	90/331 (27.2%)	89/368 (24.2%)	0.7800
Per clinical pregnancy	13/18 (72.2%)	134/170 (78.8%)	90/106 (84.9%)	89/110 (80.9%)	0.5444
**Mode of Delivery**					
Normal vaginal delivery	8/11 (72.7%)	79/120 (65.8%)	47/74 (63.5%)	32/79 (40.5%)	P5 = 0.0020 P6 = 0.0100
Cesarean section	3/11 (27.3%)	30/120 (25.0%)	21/74 (28.4%)	41/79 (51.9%)	P5 = 0.0010 P6 = 0.0100
Vacuum extraction	0/11 (0%)	11/120 (9.2%)	6/74 (8.1%)	6/79 (7.6%)	NS
Mean birthweight (g)	2734 ± 542	3005 ± 538	3095 ± 483	3156 ± 664	0.1400
Gestational diabetes	0/9 (0%)	12/117 (10.3%)	13/68 (19.1%)	14/72 (19.4%)	0.1200
Preeclampsia	2/9 (22.2%)	4/117 (3.4%)	5/68 (7.4%)	7/72 (9.7%)	0.1333
Preterm delivery (<37 wk)	2/9 (22.2%)	6/117 (5.1%)	1/68 (1.5%)	4/72 (5.6%)	0.1500
IUGR	1	3	1	2	NS
LGA	0	1	0	3	NS
IUFD	0	0	0	2	NS
Shoulder dystocia	4	1	0	0	NS
Fetal anemia	0	0	0	1	NS

Values are n (%) or mean ± SD. BMI = body mass index; wk = weeks; g = grams; IUGR = intrauterine growth restriction; LGA = large for gestational age; IUFD = intrauterine fetal demise; NS = not significant. BH—(Benjamini–Hochberg). P1 = underweight vs. normal weight, P2 = underweight vs. overweight, P3 = underweight vs. obese, P4 = normal weight vs. overweight, P5 = normal weight vs. obese, P6 = overweight vs. obese.

**Table 5:** Baseline and cycle characteristics according to clinical pregnancy status and live birth.

Clinical Pregnancy			
Variable	No Clinical Pregnancy (n = 911)	Yes—Clinical Pregnancy (n = 412)	*p*-Value
BMI (kg/m^2^)	27.0 ± 6.04	26.7 ± 6.04	0.51
Age (years)	35.4 ± 6.47	32.7 ± 5.80	<0.001
**Cause of Infertility**			
Male factor	310 (35.1%)	167 (41.0%)	0.038
Anovulation	46 (5.2%)	36 (8.8%)	0.013
Tubal/uterine factor	96 (10.9%)	40 (9.8%)	0.57
Endometriosis	15 (1.7%)	4 (1.0%)	0.46
Unexplained infertility	397 (44.9%)	153 (37.6%)	0.013
Fertility preservation	20 (2.3%)	7 (1.7%)	0.53
1st estradiol (E2, IU/L)	55.2 [35–151]	63.1 [38–267]	0.006
1st FSH (IU/L)	8.89 ± 3.44 (n = 103)	8.40 ± 5.44 (n = 32)	0.54
1st LH (IU/L)	6.05 ± 3.78	5.53 ± 4.28	0.034
Endometrial thickness (mm)	9.62 ± 2.45	10.4 ± 2.38	<0.001
No. previous transferred cycles	3.15 ± 3.09	2.62 ± 2.54	0.002
Retrieved oocytes	8.70 ± 6.00	10.0 ± 6.30	<0.001
Last estradiol (E2, IU/L)	239 [122–1041]	944 [492–1625]	<0.001
Last LH (IU/L)	3.82 ± 2.90	3.22 ± 2.35	0.014
Last progesterone (ng/mL)	0.45 [0.22–1.67]	1.09 [0.45–19.2]	<0.001
**Live birth**			
	No (n = 73)	yes (n = 325)	*p*-value
BMI (kg/m^2^)	26.5 ± 6.22	26.7 ± 6.12	0.75
BMI (kg/m^2^)			0.62
1	4 (5.5%)	13 (4.0%)	
2	33 (45.2%)	133 (40.9%)	
3	15 (20.5%)	90 (27.7%)	
4	21 (28.8%)	89 (27.4%)	
Age (years)	34.5 ± 6.32	32.3 ± 5.6	**0.002**
**Cause of Infertility**			
Male factor; n = 158	31 (43.1%)	127 (39.6%)	0.58
Anovulation; n = 35	5 (6.8%)	30 (9.2%)	0.65
Mechanical; n = 39	7 (9.6%)	32 (9.8%)	0.95
Endometriosis; n = 4	0	4 (1.2%)	1.00
Unexplained infertility; n = 150	29 (39.7%)	121 (37.2%)	0.69
Fertility preservation; n = 7	0	7 (2.2%)	0.36
1st estradiol (E2, IU/L)	58.8 [36.9–167.5]	67.9 [37.9–285.8]	0.20
1st FSH (IU/L)	9.26 [7.55–14.8]	7.26 [5.2–8.3]	**0.02**
	N = 6	N = 25	
1st LH (IU/L)	5.17 [2.85–8.01]	4.6 [2.89–7.00]	0.32
1s progesterone (ng/mL)	0.20 [0.10–0.32]	0.21 [0.12–0.33]	0.40
Endometrial thickness (mm)	10.0 ± 3.29	10.5 ± 2.08	0.18
No. previous transferred cycles	3.22 ± 2.94	2.49 ± 2.45	**0.028**
Retrieved oocytes	8.88 ± 5.96	10.4 ± 6.38	0.067
Last estradiol (E2, IU/L)	883 [350–1308]	957 [513–1673]	0.17
Last LH (IU/L)	3.17 [1.86–5.52]	2.72 [1.47–3.74]	0.11
Last progesterone (ng/mL)	1.00 [0.37–18.5]	1.11 [0.58–22.4	0.68

Values are mean ± SD, median [IQR], or n (%). BMI = body mass index; E2 = estradiol; FSH = follicle-stimulating hormone; LH = luteinizing hormone; mm = millimeters; ng/mL = nanograms per milliliter; IQR = interquartile range; SD = standard deviation.

**Table 6:** Multivariable logistic regression analysis of predictors for clinical pregnancy and live birth.

Variable	B	*p*-Value	Adjusted OR	95% CI
**Predictors for clinical pregnancy**				
BMI Category				
Normal weight; reference	—	—	—	—
Underweight	−0.607	0.467	0.545	0.106–2.802
Overweight	0.015	0.962	1.015	0.551–1.867
Obese	0.359	0.201	1.432	0.826–2.483
Age (years)	−0.036	0.074	0.965	0.927–1.003
Male factor (yes)	0.01	0.968	1.01	0.610–1.673
Last LH (IU/L)	−0.066	0.167	0.936	0.852–1.028
Last progesterone (ng/mL)	0.056	<0.001	1.057	1.038–1.077
Endometrial thickness (mm)	0.112	0.02	1.118	1.018–1.229
**Predictors for live birth**				
Age (years)	−0.078	0.016	0.925	0.868–0.986
BMI	0.024	0.353	1.024	0.973–1.078
Embryo Transfer Type		0.896		
Fresh single-embryo transfer	0.142	0.720	1.152	0.531–2.502
Fresh double-embryo transfer	−0.134	0.854	0.874	0.209–3.658
Retrieved oocytes	0.076	0.019	1.079	1.013–1.150
Endometrial thickness (mm)	0.013	0.863	1.013	0.876–1.171

BMI = body mass index; OR = odds ratio; CI = confidence interval; LH = luteinizing hormone; IU/L = international units per liter; ng/mL = nanograms per milliliter; mm = millimeters.

## Data Availability

The datasets supporting the conclusions of this article are available from the corresponding author on reasonable request.

## Supplementary Materials

The following supporting information can be downloaded at: https://www.mdpi.com/article/10.3390/jcm15062182/s1, Table S1: characteristics of fertility preservation cycles included in the cohort.
