# Supplementary material for: Beyond the Scale: Effects of Maternal Obesity on Embryo Morphokinetics and IVF Outcomes
Source: J Clin Med. 2026 Mar 12;15(6):2182. doi: 10.3390/jcm15062182 (PMC13027056; doi:10.3390/jcm15062182)
Supplement: Supplementary file 1 [file jcm-15-02182-s001.zip › jcm-4119653-supplementary.pdf]

Table S1: *Characteristics of fertility preservation cycles included in the cohort*

Characteristic	Underweight (n=14)	Normal weight (n=139)	Overweight (n=65)	Obese (n=32)	p-value
BMI (kg/m^2)	17.7±0.68	21.9±1.74	27.2±1.49	34.8±3.34	NA
Age (years)	35.4±1.26	34.8±4.27	35.2±4.94	36.6±3.36	P=0.24
Basal FSH (IU/L)†	14.0±0.42 N=2	7.24±2.1 N=32	8.38±4.10 N=24	6.79±1.69 N=8	0.017
Basal LH (IU/L)† LAST	3.39±2.35 N=10	4.30±5.60 N=91	3.64±2.08 N=46	5.14±4.72 N=23	0.58
Last Endometrial thickness (mm)	8.15±1.89	8.70±2.62	9.38±2.17	9.61±3.35	0.20
Previous IVF cycles	1.79±0.80	1.79±1.32	2.22±1.61	2.16±1.90	0.21
Total Retrieved	12.1±5.8	13.5±10.4	10.2±8.03	8.66±7.2	P[5]=0.02

Values are presented as mean ± standard deviation (SD), median [interquartile range (IQR)], or n (%). BMI = body mass index (kg/m^2); FSH = follicle-stimulating hormone; LH = luteinizing hormone; mm = millimeters; IVF = in vitro fertilization; IQR = interquartile range; SD = standard deviation.
